# Supplementary material for: Oxime-functionalized anti-insecticide fabric reduces insecticide exposure through dermal and nasal routes, and prevents insecticide-induced neuromuscular-dysfunction and mortality
Source: Nat Commun. 2024 Jun 6;15:4844. doi: 10.1038/s41467-024-49167-3 (PMC11156901; doi:10.1038/s41467-024-49167-3)
Supplement: Supplementary file 1 — Reporting Summary [file 41467_2024_49167_MOESM1_ESM.pdf]

nature portfolio

Corresponding author(s): Praveen Kumar Vemula

Last updated by author(s): 2024/05/03

Reporting Summary

Nature Portfolio wishes to improve the reproducibility of the work that we publish. This form provides structure for consistency and transparency in reporting. For further information on Nature Portfolio policies, see our Editorial Policies and the Editorial Policy Checklist.

Statistics

For all statistical analyses, confirm that the following items are present in the figure legend, table legend, main text, or Methods section.

n/a	Confirmed	
☐	☒	The exact sample size (*n*) for each experimental group/condition, given as a discrete number and unit of measurement
☐	☒	A statement on whether measurements were taken from distinct samples or whether the same sample was measured repeatedly
☐	☒	The statistical test(s) used AND whether they are one- or two-sided<br>*Only common tests should be described solely by name; describe more complex techniques in the Methods section.*
☒	☐	A description of all covariates tested
☐	☒	A description of any assumptions or corrections, such as tests of normality and adjustment for multiple comparisons
☐	☒	A full description of the statistical parameters including central tendency (e.g. means) or other basic estimates (e.g. regression coefficient) AND variation (e.g. standard deviation) or associated estimates of uncertainty (e.g. confidence intervals)
☐	☒	For null hypothesis testing, the test statistic (e.g. *F*, *t*, *r*) with confidence intervals, effect sizes, degrees of freedom and *P* value noted<br>*Give P values as exact values whenever suitable.*
☒	☐	For Bayesian analysis, information on the choice of priors and Markov chain Monte Carlo settings
☒	☐	For hierarchical and complex designs, identification of the appropriate level for tests and full reporting of outcomes
☒	☐	Estimates of effect sizes (e.g. Cohen's *d*, Pearson's *r*), indicating how they were calculated

Our web collection on statistics for biologists contains articles on many of the points above.

Software and code

Policy information about availability of computer code

Data collection	Insecticide quantification data was collected using UFLC (Shimadzu, PDA: SPD-M20A, C18 reverse phase column: LC-20AD Prominence Chromatograph). Absorption data was collected using Shimadzu, Japan. NMR data was collected on Bruker AVANCE III HD Spectrometer equipped with TCI probe. Mass spectra data was collected LCQ Fleet Mass Spectrometer (Thermo Scientific). Electromyogram data was collected using Muscle-Spiker box (Backyard Brains).
Data analysis	NMR data was analyzed using MestRenova Version 6.0.2-5475 (Mestrelab Research S. L.). UFLC data was analyzed using LabSolutions Version 5.118 (Shimadzu Corporations).

For manuscripts utilizing custom algorithms or software that are central to the research but not yet described in published literature, software must be made available to editors and reviewers. We strongly encourage code deposition in a community repository (e.g. GitHub). See the Nature Portfolio guidelines for submitting code & software for further information.

Data

Policy information about availability of data

All manuscripts must include a data availability statement. This statement should provide the following information, where applicable:

- Accession codes, unique identifiers, or web links for publicly available datasets
- A description of any restrictions on data availability
- For clinical datasets or third party data, please ensure that the statement adheres to our policy

All data generated or analysed during this study are included in this published article and its supplementary information files or are available from the

corresponding author upon request. Source data are provided with this paper.

Human research participants

Policy information about studies involving human research participants and Sex and Gender in Research.

Reporting on sex and gender	*Use the terms sex (biological attribute) and gender (shaped by social and cultural circumstances) carefully in order to avoid confusing both terms. Indicate if findings apply to only one sex or gender; describe whether sex and gender were considered in study design whether sex and/or gender was determined based on self-reporting or assigned and methods used. Provide in the source data disaggregated sex and gender data where this information has been collected, and consent has been obtained for sharing of individual-level data; provide overall numbers in this Reporting Summary. Please state if this information has not been collected. Report sex- and gender-based analyses where performed, justify reasons for lack of sex- and gender-based analysis.*
Population characteristics	*Describe the covariate-relevant population characteristics of the human research participants (e.g. age, genotypic information, past and current diagnosis and treatment categories). If you filled out the behavioural & social sciences study design questions and have nothing to add here, write "See above."*
Recruitment	*Describe how participants were recruited. Outline any potential self-selection bias or other biases that may be present and how these are likely to impact results.*
Ethics oversight	*Identify the organization(s) that approved the study protocol.*

Note that full information on the approval of the study protocol must also be provided in the manuscript.

Field-specific reporting

Please select the one below that is the best fit for your research. If you are not sure, read the appropriate sections before making your selection.

- [x] Life sciences
- [ ] Behavioural & social sciences
- [ ] Ecological, evolutionary & environmental sciences

For a reference copy of the document with all sections, see nature.com/documents/nr-reporting-summary-flat.pdf

Life sciences study design

All studies must disclose on these points even when the disclosure is negative.

Sample size	We performed at least three independent biological replicates. The data was within +/- 10% variation, and sufficient to obtain statistical significant data.
Data exclusions	No data were excluded from the analyses.
Replication	All experiments were repeated at least twice with the same conclusion. The exact number of experiment replications are noted in the figure legends.
Randomization	All samples were randomly assigned to groups.
Blinding	For in vivo experiments, the investigators were blinded to the group allocation during the data collection.

Reporting for specific materials, systems and methods

We require information from authors about some types of materials, experimental systems and methods used in many studies. Here, indicate whether each material, system or method listed is relevant to your study. If you are not sure if a list item applies to your research, read the appropriate section before selecting a response.

Materials & experimental systems

n/a	Involved in the study	
x		Antibodies
x		Eukaryotic cell lines
x		Palaeontology and archaeology
	x	Animals and other organisms
x		Clinical data
x		Dual use research of concern

Methods

n/a	Involved in the study	
x		ChIP-seq
x		Flow cytometry
x		MRI-based neuroimaging

Animals and other research organisms

Policy information about studies involving animals; ARRIVE guidelines recommended for reporting animal research, and Sex and Gender in Research

Laboratory animals	Sprague-Dawley albino rats were used which were housed in the institute animal facility. All studies were performed with both male and female rats between 10 to 14 weeks of age. Rats were housed in facility that was subjected to 12/12 hours light/dark cycle, at a temperature of 20-25 oC and humidity of 35-65%.
Wild animals	No wild animals were used in these studies.
Reporting on sex	Sex was not considered in this study. Both, male and female rats were used.
Field-collected samples	The current study did not use utilize field-collected samples.
Ethics oversight	The experimental protocols were approved by the Institutional Animal Ethics Committee (IAEC) at the Institute for Stem Cell Science and Regenerative Medicine (INS-IAE-2020/15(R1)), and were in accordance with the guidelines from the Committee for the Purpose of Control and Supervision of Experiments on Animals (CPCSEA), Govt. of India.

Note that full information on the approval of the study protocol must also be provided in the manuscript.
