# Supplementary material for: Oxime-functionalized anti-insecticide fabric reduces insecticide exposure through dermal and nasal routes, and prevents insecticide-induced neuromuscular-dysfunction and mortality
Source: Nat Commun. 2024 Jun 6;15:4844. doi: 10.1038/s41467-024-49167-3 (PMC11156901; doi:10.1038/s41467-024-49167-3)
Supplement: Supplementary file 2 — Supplementary Information [file 41467_2024_49167_MOESM2_ESM.pdf]

Supplementary Information for -

Oxime-functionalized anti-insecticide fabric reduces insecticide exposure through dermal and nasal routes, and prevents insecticide-induced neuromuscular-dysfunction and mortality

Mahendra K Mohan[1], Ketan Thorat[1], Theja Parassini Puthiyapurayil[1], Omprakash Sunnapu[2], Sandeep Chandrashekharappa[1], Venkatesh Ravula[1], Rajamohammed Khader[1], Aravind Sankaranarayanan[1,3], Hadi Muhammad[1], and Praveen Kumar Vemula[1,*]

1. Institute for Stem Cell Science and Regenerative Medicine (DBT-inStem), GKVK Post, Bellary Road, Bangalore 560065, Karnataka, India

2. Sepio Health Private Limited, Bangalore, 560065, Karnataka, India

3. Tata Institute for Genetics and Society (TIGS), inStem, GKVK Post, Bellary Road, Bangalore 560065

Corresponding author:

*e-mail: praveenv@instem.res.in

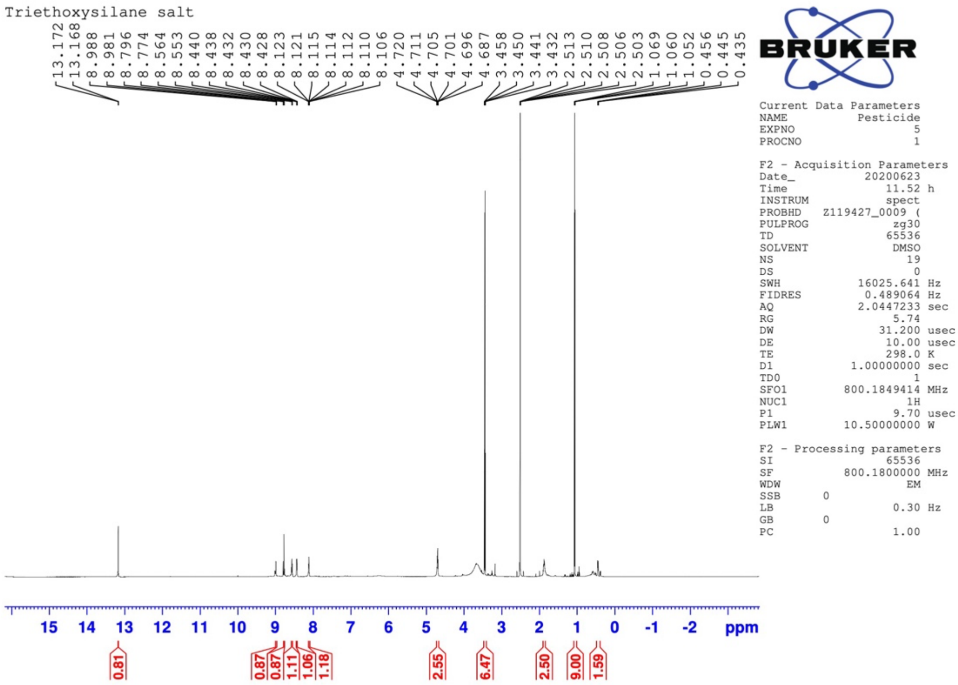

Supplementary Figure S1. Characterization of silyl-oxime. ^{1}H-NMR spectra of 3(pyridine-2-aldoxime)propyl-triethoxysilane in DMSO-D_6 which was recorded using 800 MHz machine.

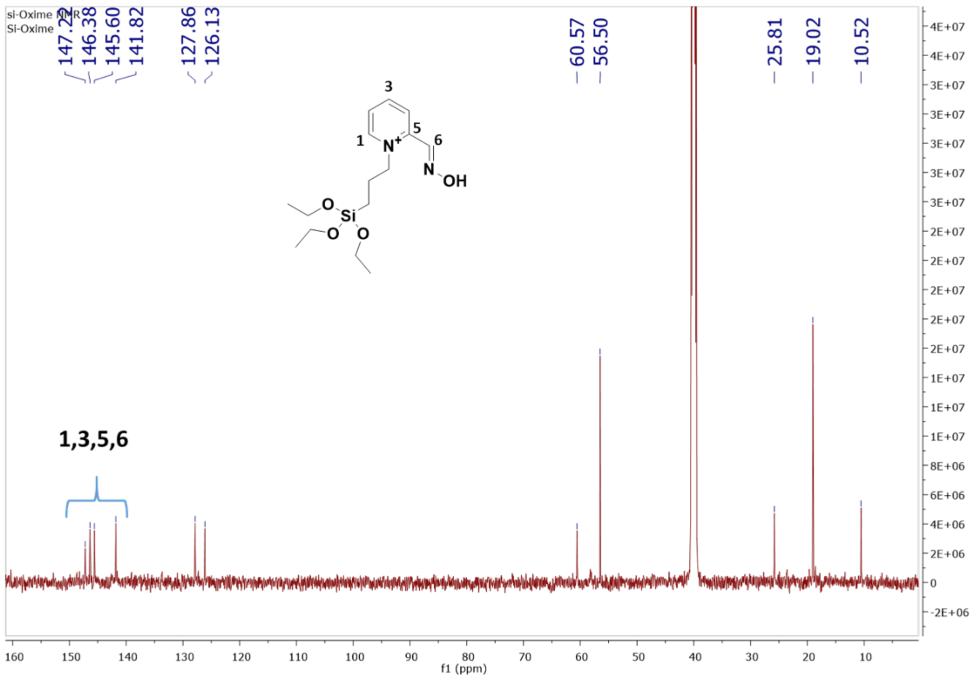

Supplementary Figure S2. Characterization of silyl-oxime. ^{13}C-NMR spectra of 3(pyridine-2-aldoxime)propyl-triethoxysilane in D_2O which was recorded using 800 MHz machine.

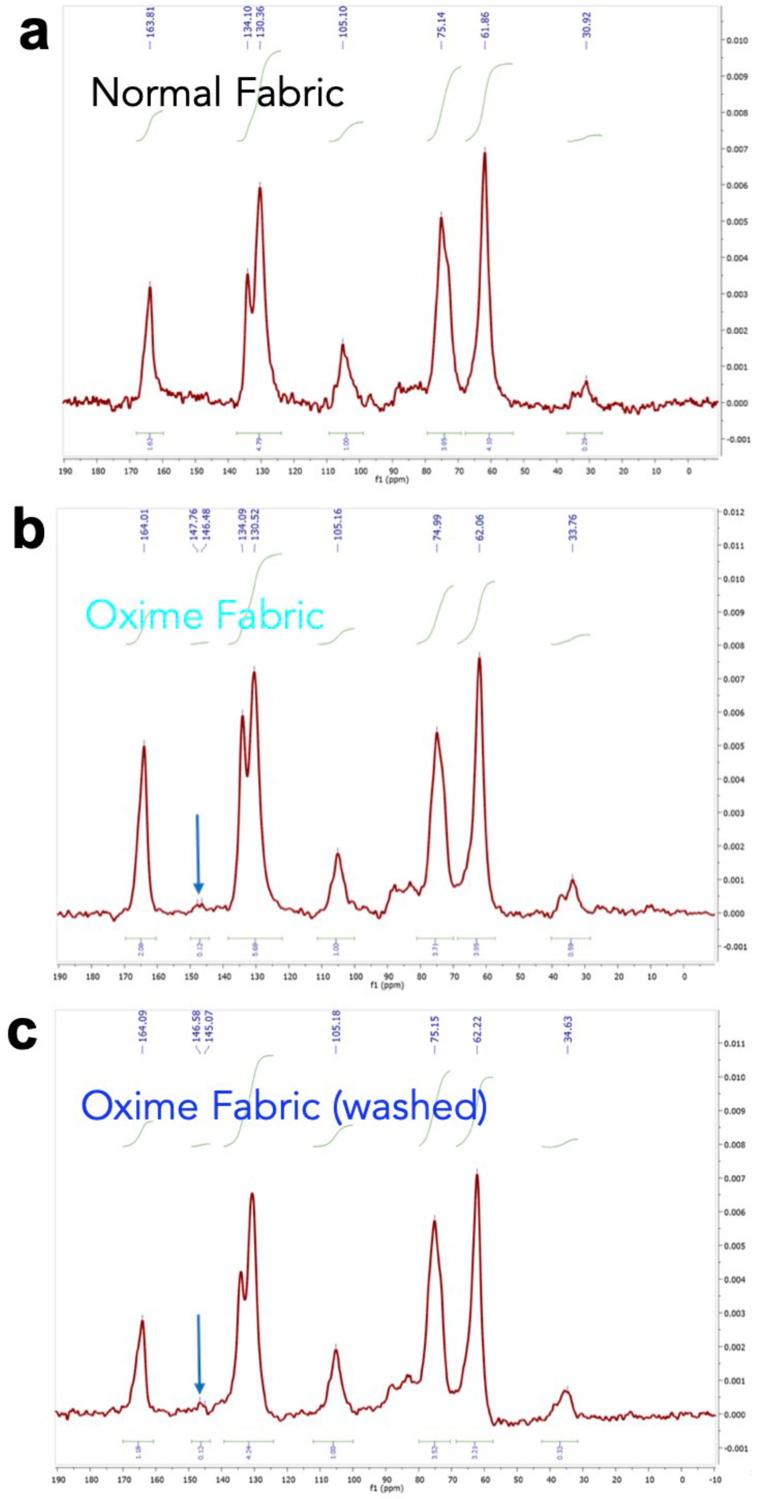

Supplementary Figure S3. Solid state Cross Polarization Magic Angle Spinning (CP/MAS) ^{13}C-NMR spectra. Spectra of **a**, Normal Fabric, **b**, Oxime Fabric after curing, and **c**, Oxime Fabric after extensive washing. Peaks corresponding to pyridinum carbons (blue

arrow) were present only on Oxime-fabric and they remain present after washing suggesting the presence of silyl-oxime on Oxime Fabric.

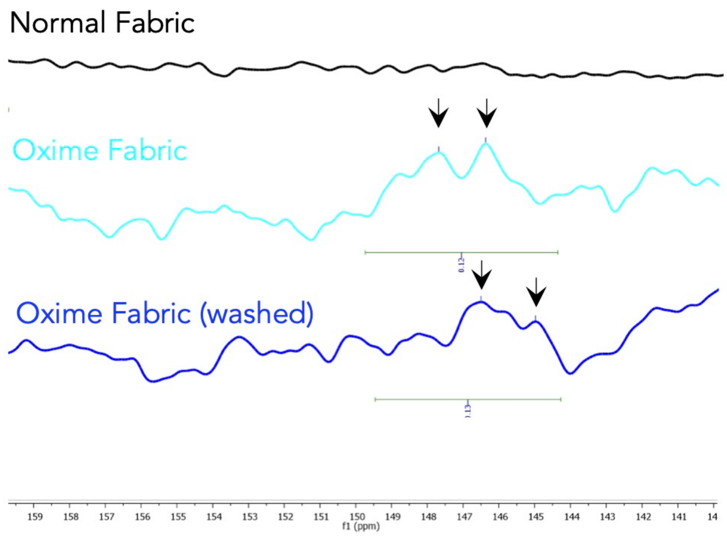

Supplementary Figure S4. Solid state Cross Polarization Magic Angle Spinning (CP/MAS) ^{13}C-NMR spectra. Spectra of **a**, Normal Fabric, **b**, Oxime Fabric after curing, and **c**, Oxime Fabric after extensive washing. Peaks corresponding to pyridinum carbons (blue arrow) were present only on Oxime-fabric and they remain present after washing suggesting the presence of silyl-oxime on Oxime Fabric. (Selective region of Supplementary Fig. S3).

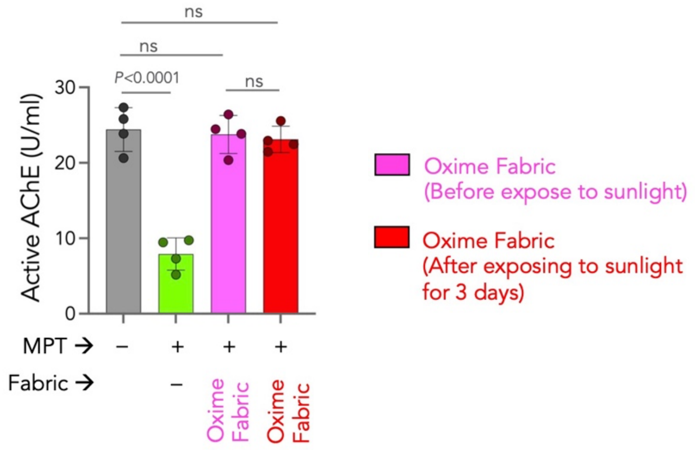

Supplementary Figure S5. Oxime-fabric retains its activity after exposed to sunlight for three days, *ex vivo*. The stability of Oxime-fabric upon exposure to sunlight has been tested. The efficacy of Oxime-fabric to prevent insecticide-induced AChE inhibition, before and after exposed to sunlight for three days remained the same, which suggests that Oxime-fabric could be used under sunlight as farmers do. Data are mean ± s.d. (n = 4, from independent

experiments). *P* values were determined by ordinary one-way ANOVA with Tukey's post hoc analysis by GraphPad PRISM 9, and exact P values are indicated. ns = not significant. Source data are provided as a Source Data file.

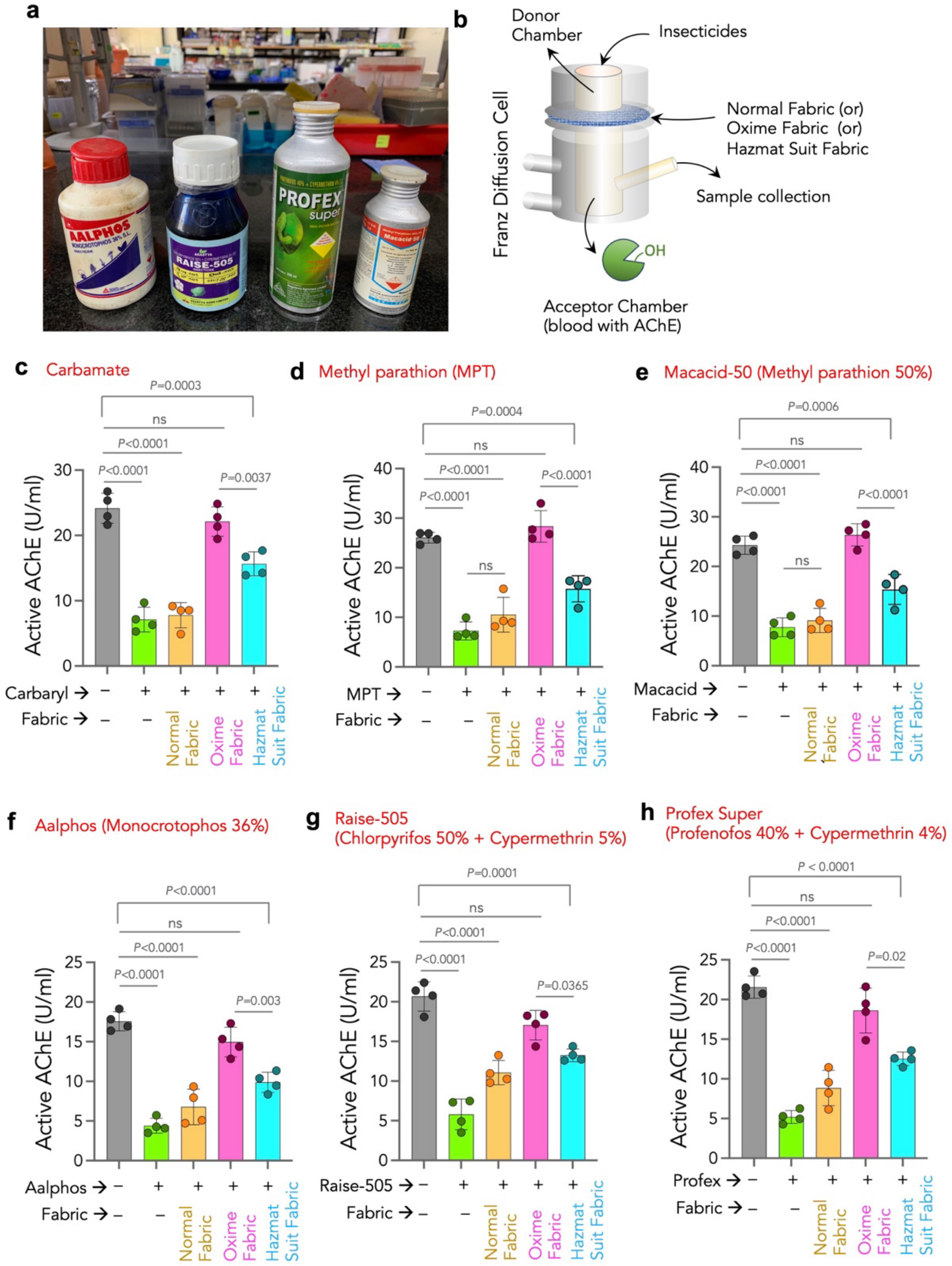

Supplementary Figure S6. Oxime-fabric deactivates commercial insecticide formulations, and prevents AChE inhibition, *ex vivo*. a, Photograph image of commercial formulations used in this study. **b**, The efficacy of Oxime-fabric to prevent commercial insecticide formulations-induced AChE inhibition, an *ex vivo* assay was performed using rat

blood. AChE containing rat blood was placed in the acceptor chamber. In the donor chamber, various insecticides such as carbamate (**c**), MPT (**d**), Macacid-50 (**e**), Aalphos (**f**), Raise-505 (**g**), and Profex Super (**h**) were added in the presence of normal fabric or Hazmat suit fabric or Oxime-fabric. Active AChE was measured in unexposed native blood and 3 hr post addition of pesticide formulations in these groups. The normal fabric and Hazmat suit fabric could not prevent diffusion of commercial pesticide formulations into the acceptor chamber, which resulted in significant inhibition of AChE activity. On the contrary, Oxime-fabric could hydrolyze carbamate and insecticides in commercial formulations before they diffuse, hence prevented the insecticide-induced inhibition of AChE. Data are mean ± s.d. (n = 4, from independent experiments). For **c**-**h**, P values were determined by ordinary one-way ANOVA with Tukey's post hoc analysis by GraphPad PRISM 9, and exact P values are indicated. ns = not significant. Source data are provided as a Source Data file.

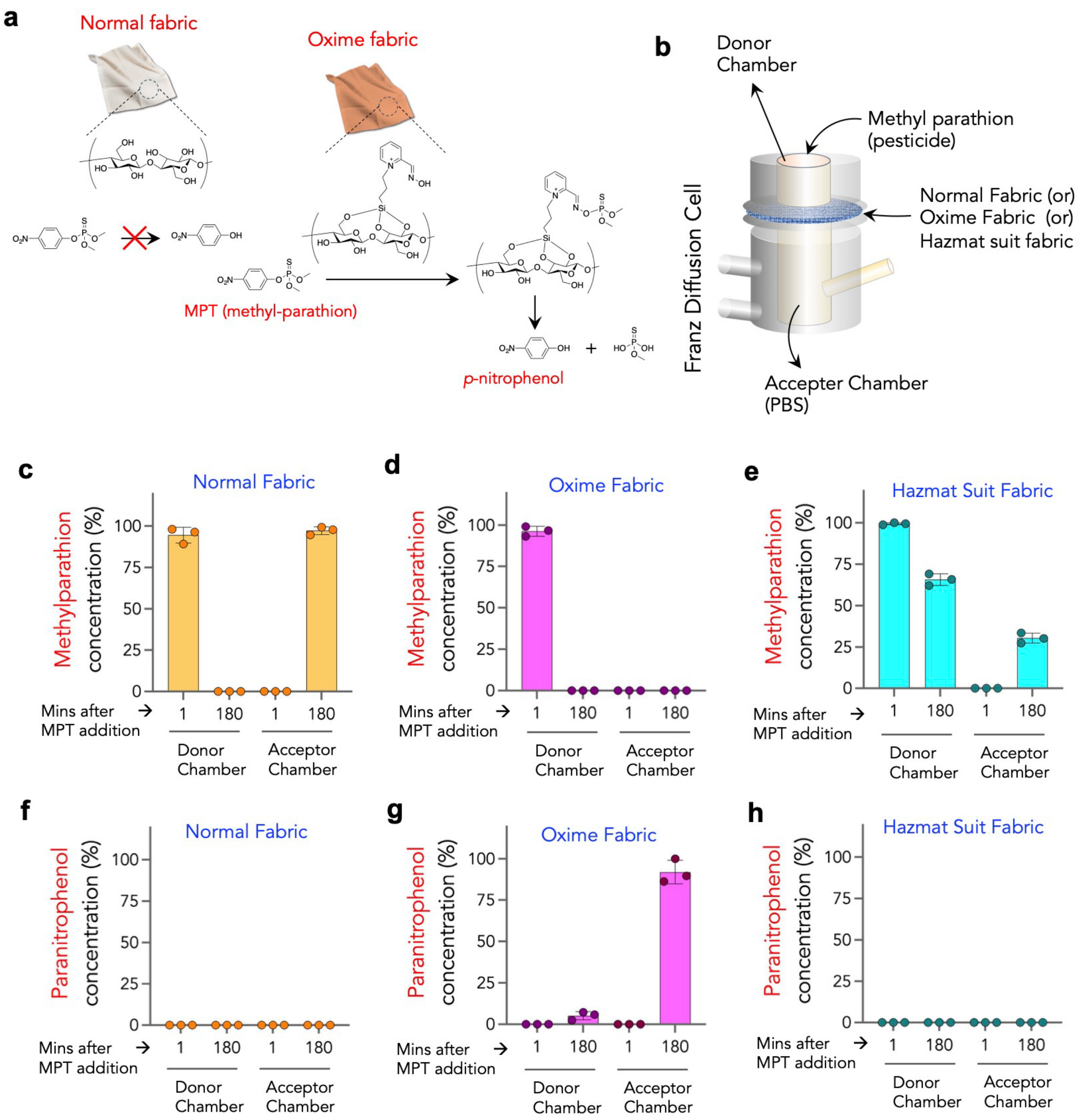

Supplementary Figure S7. Oxime-fabric chemically deactivates organophosphate insecticides by hydrolysis, *in vitro*. a, Chemical scheme for the hydrolysis of organophosphate insecticide, MPT, by Oxime-fabric. **b**, Schematic of Franz diffusion cell, either normal fabric or Hazmat suit fabric or Oxime-fabric was placed on a dialysis membrane which was placed between donor and acceptor chambers. **c-h**, Concentration of organophosphate ester methyl parathion (MPT) and para-nitrophenol (hydrolytic degradation product of methyl parathion) in donor and acceptor chambers was measured using UFLC. The presence of normal fabric did not prevent the diffusion of MPT into the acceptor chamber and

could not hydrolyze MPT to generate para-nitrophenol, whereas Oxime-fabric quantitatively hydrolyzed to prevent the penetration of toxic MPT into the acceptor chamber. Hazmat suit fabric provided moderate barrier function, but could not prevent pesticide diffusion. Approximately, 30% of methyl-parathion was diffused into acceptor chamber, and Hazmat suit fabric could not hydrolyse MPT, hence, could not form para-nitrophenol. Data are mean ± s.d. (*n* = 3, from independent experiments). Source data are provided as a Source Data file.

Cost analysis of Oxime-fabric PPEs and comparison with commercially available PPEs.

We have done a detailed production cost analysis to manufacture each PPE suit, including bulk scale production of active components, cost for textile processing, and overall production cost, labour cost, and tentative pricing of the final product. A tentatively proposed price for Oxime-Fabric PPE is calculated as USD 18 for each suit. Thus far, we have already completed the production of 1000 scale Oxime-Fabric PPEs. The cost provided is based on 1000 suit-scale production, which could be even reduced further when scale reaches >100,000 suits.

In **Supplementary Table S1**, a detailed cost comparison has been provided. Prices of various PPEs from the USA, UK, Singapore, and Australia were given. Interestingly, none of the existing PPEs can chemically deactivate pesticides. They are disposable physical barrier suits. Depending on the type of crops used and farming seasons, these suits will be used an average of 60 to 90 times, annually. Therefore, given the nature of the reusability of Oxime-Fabric PPEs, either only one or a maximum of two suits will be sufficient for a year, whereas except for two commercial PPE kits, the rest of them are non-reusable/disposable PPEs. Hence, the annual budget for these PPEs was calculated accordingly. This analysis suggests the cost-economic nature and affordability of Oxime-Fabric PPEs.

Supplementary Table S1. Cost comparison between Oxime-Fabric PPE *vs* commercially available PPEs. Unit prices for these suits were given. Additionally, based on the annual usage of PPEs, the projected annual budget has been estimated.

**Price comparison with existing PPEs**				
				**Annual Budget for PPEs**
	**USD**	**Pesticide Deactivation**	**Reusability**	**USD**
**Oxime-fabric**	**18**	**Yes**	**Yes**	36
Basic-Safety Kit (USA)	25	No	No (disposable)	1500 to 2250
Tiger Tough PPEs	13	No	No (disposable)	780 to 1170
Hazmat suits	10	No	No (disposable)	600 to 900
Medtecs Hazmat suits	14	No	No (disposable)	840 to 1260
Pentagon Safety equipment	21	No	No (disposable)	1260 to 1890
Progreen Suits (UK)	31	No	No (disposable)	1860 to 2790
DuPont Tyvek	48	No	Partial resuable	2880 to 4320
RS Pro Blue (Singapore)	8	No	No (disposable)	480 to 720
3M Pesticide Kit	104	No	Partial resuable	6240 to 9360
**Average times farmers use the suit yearly**	60-90 times			

Therefore, we can confidently claim that Oxime-Fabric PPE is cheap for mass-production, and it is an affordable solution that may protect millions of agriculture workers worldwide.
